# Gut Microbiome Health in Farm Animals and Fish: Implications for Human Health and the Risk of Gastrointestinal Diseases

**DOI:** 10.3390/microorganisms14020447

**Published:** 2026-02-12

**Authors:** Andrada Ihuț, Camelia Răducu, Mirela Ranta, Andreea Andrecan, Paul Uiuiu

**Affiliations:** 1 Department of Technological Sciences, Faculty of Animal Science and Biotechnologies, University of Agricultural Sciences and Veterinary Medicine Cluj-Napoca, 3-5 Mănăstur Street, RO-400372 Cluj-Napoca, Romania; ihut.andrada@usamvcluj.ro; 2 Department of Plant Culture, University of Agricultural Sciences and Veterinary Medicine Cluj-Napoca, 3-5 Mănăstur Street, RO-400372 Cluj-Napoca, Romania; 3 Department of Horticulture and Landscaping, Faculty of Horticulture and Rural Development Business, University of Agricultural Sciences and Veterinary Medicine Cluj-Napoca, 3-5 Mănăstur Street, RO-400372 Cluj-Napoca, Romania; andreea.andrecan@usamvcluj.ro; 4 Department of Fundamental Sciences, Faculty of Animal Science and Biotechnologies, University of Agricultural Sciences and Veterinary Medicine Cluj-Napoca, 3-5 Mănăstur Street, RO-400372 Cluj-Napoca, Romania; paul.uiuiu@usamvcluj.ro

**Keywords:** one health approach, microbiome diversity, dysbiosis, zoonotic pathogens, microbial transmission, antimicrobial resistance

## Abstract

The gut microbiome is central to immune, metabolic, and gastrointestinal health across species. Dysbiosis disrupts microbial communities and is linked to inflammatory bowel disease, celiac disease, and other immune-mediated gastrointestinal disorders. This review addresses the central question of how diet- and environment-driven gut dysbiosis in farm animals and fish is transmitted through the food chain to influence human gastrointestinal health within a One Health framework. This review synthesizes recent evidence within the One Health framework, focusing on how diet- and environment-induced dysbiosis in farm animals and fish can influence human gastrointestinal health via the food chain. We highlight mechanisms of immune modulation, alterations in food products, and the risks of pathogen transmission and antimicrobial resistance. An important limitation of the current body of evidence is the lack of studies that comprehensively trace the proposed axis from animal gut dysbiosis to human health outcomes. Emerging interventions, including precision nutrition, probiotics, and microbiota-targeted therapies, show potential for restoring microbial balance, though translating these findings into clinical practice remains challenging. By integrating human, veterinary, and environmental perspectives, this work proposes a novel cross-species microbiome–diet–immune framework to guide future research and interventions, advancing One Health strategies for disease prevention, antimicrobial resistance mitigation, and sustainable gastrointestinal health.

## 1. Introduction

Defining a healthy microbiome remains a major challenge today, given the high compositional variability between individuals. It is impossible to establish a fixed set of microorganisms as a universal marker of health [1,2,3]. Instead, accumulating evidence suggests that a healthy microbiome should be viewed as a dynamic functional spectrum [4], characterized by microbial diversity, functional stability, and the preservation of essential community roles, including nutrient metabolism, immune modulation, and protection against pathogenic colonization. These functional attributes, rather than the presence or absence of specific microbial taxa, are increasingly recognized as more reliable indicators of gut health across species [3,5,6,7,8,9].

**Figure 1:**
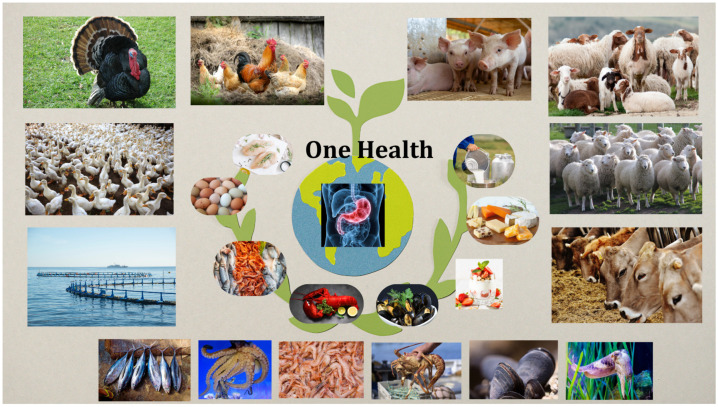
One World–One Health: Interconnections among humans, livestock, and the environment. The image was created using Canva.

Rather than being a passive microbial community, the gut microbiome functions as an immunological interface that influences intestinal health in both humans and food-producing animals. A healthy gut microbiome plays a crucial role in preventing and modulating gastrointestinal diseases by maintaining local immune balance [10]. The most important mechanisms by which the microbiota influence immunity include producing metabolites such as transformed bile acids and n-butyrate, which regulate macrophage function by inhibiting histone deacetylases (HDACs) [11]; stimulating or inhibiting immune cells (Tregs, Th17, macrophages, and neutrophils) [12]; and controlling inflammation through cytokines and antigens presented by dendritic cells [13,14]. When this functional equilibrium is disrupted, alterations in microbial metabolic output and barrier integrity can lead to increased intestinal permeability and sustained immune activation. These mechanisms are directly involved in the pathogenesis of inflammatory bowel disease (IBD) [15,16], coeliac disease [17], and other immunologically based digestive disorders [18]. Beyond bacterial dysbiosis, increasing evidence indicates that the gut virome, particularly bacteriophages, is a critical regulator of microbial community structure and mucosal immune responses, with a documented role in the pathogenesis of IBD [19]. Beyond bacteria, the gut microbiome also includes viruses (the virome) and fungi (the mycobiome), which interact closely with bacterial communities and the host’s immune system [20]. Emerging evidence shows that these non-bacterial components play key roles in shaping the microbial ecosystem, regulating immune responses, and supporting metabolic functions [21]. Although important, they are often overlooked in microbiome studies, leaving gaps in our understanding. Considering the virome and mycobiome helps provide a more complete picture of how the microbiome affects health and disease across different species [22]. Farm animals and fish constitute a primary source of human nutrition, providing meat, dairy, eggs, and fish-derived products [23,24]. These species are frequently exposed to a range of stressors, including suboptimal husbandry conditions, nutritional factors, antibiotic administration, and environmental challenges. These stressors can perturb the intestinal microbial community, leading to dysbiosis, compromised gut barrier function, and disrupted metabolic and immune function. Dysbiosis can also alter host immune responses and trigger intestinal inflammation, which may in turn modify the microbial composition of animal-derived food products. Consequently, these microbial imbalances have potential downstream effects on human health through the food chain, including pathogen transmission, contamination of animal products, and the spread of antimicrobial resistance [25,26]. This highlights the importance of nutrition in shaping gut microbiome composition and function, with direct consequences for host immune, metabolic, and neurological health [27,28]. To better understand these cross-species microbial interactions, it is necessary to adopt a microbiome–diet–immune axis across species. This conceptual framework traces the trajectory from the gut microbiome of food-producing animals, through diet- or environment-induced dysbiosis, to immune modulation with downstream effects on the microbial and metabolic characteristics of animal-derived food products. Ultimately, these changes may expose the human gut microbiome to metabolic and immunological perturbations, with potential consequences for gastrointestinal health. This interaction is investigated in the context of One Health, where human, animal, and environmental health are viewed as interconnected elements [29,30]. The interdependence between humans, animals, and the environment, as illustrated by the One World–One Health concept, is schematically represented in Figure 1.

The One World–One Health concept promotes human and animal welfare by integrating human medicine, veterinary medicine, and environmental sciences to address global issues such as zoonoses, food safety, and climate change [31,32,33,34]. Implementing this concept provides an interdisciplinary research framework to optimize ecosystem stability and balance. This is achieved by identifying complex relationships among biological, ecological, and anthropogenic factors and by developing rigorous forecasts for disease prevention based on a thorough understanding of cause and effect. ct [35,36,37].

This review synthesizes recent mechanistic and translational evidence on gut microbiome health in farm animals and fish within the One Health framework, highlighting the interconnected roles of diet, microbial composition, and immune regulation across species. It examines key factors influencing intestinal homeostasis and immune-mediated gastrointestinal disorders, as well as interventions to restore microbial homeostasis. It also highlights risks such as antimicrobial resistance, zoonotic pathogens, and the indirect effects of dysbiosis. Integrating perspectives from different disciplines is essential for understanding and managing health at the species and ecosystem levels. The review also examines how microbiome perturbations in food-producing animals may affect human gut health through the consumption of animal-derived products. In addition, the review critically discusses emerging microbiome-targeted interventions to restore microbial balance, as well as ongoing challenges related to antimicrobial resistance, zoonotic pathogen transmission, and the indirect consequences of dysbiosis. By integrating perspectives from human medicine, veterinary science, and environmental research, this work proposes a cross-species conceptual framework linking animal dysbiosis to human gastrointestinal disease risk, thereby advancing the One Health agenda and informing future preventive and therapeutic strategies.

## 2. Materials and Methods

This review was conducted following a structured literature search focused on the gut microbiome in farm animals and fish, with an emphasis on translational implications for human health within the One Health framework. Searches were conducted in key peer-reviewed databases, including Google Scholar, PubMed, Web of Science, and Scopus, using keywords such as ‘One Health approach’, ‘gut microbiome ‘, ‘dysbiosis’, ‘zoonotic pathogens’, ‘microbial transmission’, and ‘antimicrobial resistance’. Eligibility criteria included original research and review articles published in English between 2000 and 2026. Exclusion criteria were non-peer-reviewed sources, non-English publications, and studies without original data. The search was limited to the Title, Abstract, and Author Keywords fields. Included studies addressed gut microbiota composition, immune-mediated dysbiosis, microbial metabolites, or interventions targeting microbial balance. They followed a cross-species microbiome-diet-immune axis, tracing the trajectory from the gut microbiome of food-producing animals, through diet- and environment-induced dysbiosis, to immune modulation and consequent alterations in the microbial and metabolic composition of animal-derived food products, with potential implications for the human gut microbiome. Data synthesis focused on determinants of microbial balance, immune and systemic consequences of dysbiosis, zoonotic and antimicrobial resistance risks, and microbiome-targeted interventions. By integrating human, veterinary, and environmental perspectives, the review adopts a One Health approach to advance understanding of the gut microbiome and its relevance for disease prevention and public health.

## 3. The Intestinal Microbiome in Farm Animals and Fish: Implications for Human Health

The gut microbiome of farm animals and economically significant aquatic species is essential for maintaining health, supporting growth and production performance, and regulating feed intake [38,39]. It also influences behavior and stress responses [40] and contributes to the safety and quality of animal-derived food products [41,42,43]. Imbalances in the microbiome of farm animals can lead to reduced disease resistance and excessive antibiotic use, increasing the risk of antimicrobial resistance, a phenomenon that directly affects human health [44]. In aquaculture, the fish intestinal microbiome contributes to efficient feed conversion, resilience to pathogens, and product quality. From a human perspective, interactions with the animal microbiome primarily occur through food consumption [29,45], but can also occur through bioaerosols (e.g., farm dust) [46], direct contact during animal handling [47], and gene transfer (resistome) [48]. Therefore, maintaining a balanced intestinal microbiome in animals can mitigate contamination of animal products with pathogens and reduce the likelihood of transmitting antibiotic resistance genes. For example, the rumen microbiome directly influences milk or meat quantity and quality, animal health, and greenhouse gas emissions [49].

In accordance with the One Health principle, modern management strategies such as the use of prebiotics, probiotics, synbiotics, postbiotics, and phytobiotics [50,51,52,53,54,55] in animal nutrition not only do they improve animal health but also ensure safer, more nutritious animal products for human consumption while reducing greenhouse gas emissions.

To provide a structured analysis of species-specific differences, the following subsections examine the gut microbiomes of farm animals and fish, highlighting features of a healthy microbiome, stressors that can induce dysbiosis, and the effects on the safety and quality of animal-derived products. This analysis is framed within a cross-species perspective, linking animal gut health to human health through food, environmental exposure, and One Health considerations.

### 3.1. Ruminants

The gut microbiota of ruminants, such as cows, goats, and sheep, comprises a complex ecosystem of bacteria, protozoa, fungi, and archaea that facilitate the digestion of fibrous plants through microbial fermentation processes [56]. This ability makes ruminants unique, as they are capable of efficiently converting carbohydrates from plant cell walls into nutrients for meat and milk production [9,57].

In cows, for example, bacteria constitute about 90% of the gastrointestinal tract (GIT) microbial community and play a pivotal role in feed digestion and host metabolism. They ferment complex carbohydrates, such as cellulose, hemicellulose, and starch, producing volatile fatty acids (VFAs) that serve as the animal’s primary energy source. The main bacterial phyla are *Firmicutes* (*Bacillota*), including families such as *Ruminococcaceae*, *Lachnospiraceae*, and *Clostridiaceae*, and *Bacteroidetes*, including families such as *Bacteroidaceae* and *Rikenellaceae* [49,58]. Other phyla present in smaller quantities include *Proteobacteria*, *Actinobacteria*, *Spirochaetes*, and *Tenericutes* [59,60]. Rumen protozoa, unicellular and ciliates (*Ciliophora*), play a vital role in carbohydrate digestion in the rumen and indirectly contribute to protein metabolism by preying on bacteria and fungi. These microorganisms produce characteristic enzymes that are uncommon in other organisms, including pectin esterases (for pectin degradation), cathepsins (proteases), and various glycosyl hydrolases [61]. Taxonomically, protozoa are divided into entodinomorphs, the most abundant, which include species such as *Entodinium*, *Isotricha*, and *Dasytricha*, and *Holotrichs*. Although *Holotrichs* are less common, they contribute significantly to fiber digestion and microbial population regulation [62]. The rumen microbiome of cows comprises diverse anaerobic fungi from the *Neocallimastigomycota* phylum, which play a key role in digesting plant fibers and in the host’s metabolism [63]. *Archaea* are anaerobic prokaryotic microorganisms that play a central role in rumen methanogenesis, using hydrogen and carbon dioxide (CO_2_) to produce methane [49,64]. This process occurs through three primary metabolic pathways: hydrogenotrophic, methylotrophic, and aceticlastic. These pathways help maintain redox balance in the rumen and eliminate excess hydrogen generated by microbial fermentation [65]. Methanogenic archaea represent approximately 4% of the microbial biomass in the rumen and include species such as *Methanobrevibacter* and *Methanosarcina* [66,67].

The rumen microbiome of ruminants can undergo significant changes under conditions of physiological stress (e.g., transport or exposure to extreme temperatures) [68], inadequate nutrition (e.g., diets rich in concentrates and low in fiber) [69], or drug treatments [70].

Stressors affect the intestinal microbiome in cattle, leading to dysbiosis, altered bacterial diversity, increased abundance of pathogenic organisms, and other changes. For example, heat stress in lactating cows significantly alters the composition of the ruminal microbiome, particularly bacterial and protozoal communities [71]. Heat-sensitive breeds, such as Holstein cattle, exhibit a significantly higher number of unique operational taxonomic units (OTUs) compared with more heat-tolerant breeds. Moreover, heat stress is associated with an increased relative abundance of *Bacteroidetes* and a concomitant reduction in *Firmicutes*, resulting in an altered Firmicutes-to-Bacteroidetes ratio and the development of ruminal microbial dysbiosis [71]. Elevated ambient temperature adversely affects ruminal fermentation by lowering ruminal pH, inhibiting fibrolytic bacteria, and reducing acetate concentration, a major short-chain fatty acid (SCFA). Furthermore, heat stress significantly impairs milk quality by decreasing antioxidant capacity and altering the milk microbiota, favoring taxa associated with spoilage [72].

Inadequate maintenance and husbandry conditions of dairy cows represent another risk factor that leads to reduced milk quality and the development of mastitis, which subsequently results in oxidative stress, increased somatic cell count (SCC), decreased immunity, dysbiosis, tissue damage, and elevated levels of pro-inflammatory cytokines: IL-1β (Interleukin-1 beta), IL-6 (Interleukin-6), and TNF-α (Tumor Necrosis Factor alpha) [73,74].

Johne’s disease represents another risk factor for both animals and humans through the consumption of milk and dairy products derived from infected animals. The disease is caused by infection with *Mycobacterium avium* subsp. paratuberculosis, which is transmitted primarily via the fecal–oral route and is able to persist both in the environment and within the host organism. The infection leads to intestinal inflammation and progressive clinical manifestations following a prolonged latent period, resulting in malabsorption, diarrhoea, and weight loss [75]. The disease affects immune responses through activation of a pro-inflammatory T helper 1 (Th1) immune response and induces metabolic alterations, particularly in lipid metabolism, in animals such as cattle, sheep, and goats [75].

Overall, these effects impact animal health and food safety (Table 1). Exposure of dairy cows to thermal stress negatively affects milk quality, leading to lower concentrations of milk fat and protein. Moreover, heat stress is linked to increased somatic cell counts (SCCs) [76], reflecting compromised immune function and a higher prevalence of mastitis. The subsequent use of antimicrobial treatments raises concerns about food safety and public health, particularly regarding the emergence and spread of antimicrobial resistance (AMR). Unhygienic conditions on farms, particularly during milking, transport, and milk processing, present a significant risk to human food safety, potentially leading to illnesses ranging from enterocolitis to hemorrhagic colitis and hemolytic uremic syndrome [77,78].

Consumption of milk from MAP-infected cows has been associated with Crohn’s disease and dysbiosis in humans. The risk is particularly significant when milk is not tested for MAP and when manure from infected animals is applied as fertiliser to soil. MAP can persist in the environment for up to 12 months, enabling contamination of pasture and potential infection of healthy animals [79]. Following the One Health approach, careful monitoring of dairy herds, manure management, and soil contamination is essential to reduce the risk of zoonotic transmission.

Recent studies suggest that administering biotics (probiotics, prebiotics, postbiotics, and synbiotics) may be an effective way to maintain intestinal balance and microbiota, thereby improving animal health and performance whilst reducing the need for antibiotics [80,81]. To mitigate heat stress in dairy cows, it is recommended to implement early detection and monitoring, effective environmental management through the provision of natural and artificial shade, optimized ventilation systems, dietary adjustments and supplementation (including minerals, vitamins, and antioxidants), fiber optimization, proper design and placement of housing facilities, and genetic selection of breeds with enhanced heat tolerance [71,72,82].

Beyond strategies to reduce heat stress, additional preventive measures are necessary to manage other health challenges in dairy cows, such as mastitis, which can affect milk quality, immunity, and overall productivity. Effective mastitis prevention depends primarily on proper barn and milking hygiene [83], isolation of infected cows, prevention of overcrowding, and adequate ventilation [84]. Additional critical strategies include regular monitoring and early diagnosis, vaccination, genetic selection, and ensuring appropriate nutrition for the animals [85]. Besides the previously mentioned methods, incorporating biotics into the cows’ diet represents another effective approach. Thus, several studies [86,87] highlighted that probiotics can serve as an alternative to antibiotics for the prevention of mastitis. Specifically, oral administration of the probiotic *Bacillus subtilis* C-3102, *Saccharomyces cerevisiae* and *L. lactis* strain was associated with a significant reduction in mastitis incidence, a decrease in the average number of days of discarded milk, lower SCC, reduced levels of stress markers such as cortisol and Thiobarbituric Acid Reactive Substances (TBARS), and an improved regulation of the adaptive immune response, including an increase in CD4+ T cells and dendritic cells. The administration of probiotics via intramammary infusion, including *Enterococcus mundtii* H81, *Lactococcus lactis* DPC 3147, *Lactobacillus rhamnosus* GG, *Enterococcus faecium* SF68, *Bifidobacterium breve*, *Lactococcus lactis* subsp. *lactis* CRL1655, and *Lactobacillus perolens* CRL1724, has been shown to reduce pro-inflammatory cytokines, inhibit pathogen colonization, decrease SCC, and reduce neutrophil infiltration and inflammation [87].

**Table 1:** Impact of Cattle Gut Microbiome Disruption: Causes, Effects on Food Products, and Implications for Human Health.

Cause	Microbiome Effects	Immune/Metabolic Changes	Impact on Animal Products	Human Health Impact	One Health Prevention/Intervention Strategies	Reference
Heat stress	↑ *Bacteroidetes* ↓ *Firmicutes* Unique OTUs	↓ SCFA ↓ Ruminal pH Disturbed fermentation; Inflammation;	↓ milk production ↓ protein ↓ fat ↑ SCC	↓ milk quality ↓ milk UFA ↓ milk CLA ↓ milk flavor ↓ antioxidant capacity AMR	Genetic selection of cattle for more resistance to heat. Providing natural or artificial shade. Optimal ventilation Dietary adaptation Supplement Administration (Antioxidants, Minerals, Functional Additives)	[71,72,82,88,89,90,91]
Dysbiosis Unsanitary Conditions Mastitis	↑ *Proteobacteria* ↓ *Firmicutes* ↑ *Staphylococcus aureus*, ↑ *Streptococcus agalactiae*, ↑ *Escherichia coli* (Shiga toxin), ↑ *Streptococcus dysgalactia*, ↑ *Mycoplasma* spp., ↑*Klebsiella* ↑*Streptococcus dysgalactia*, ↑*Corynebacterium bovis*	Inflammation ↑ IL-1β, IL-6, TNF-α Oxidative stress ROS	↑ SCC ↓ milk production ↓ milk quality	AMR Hemorrhagic colitis Hemolytic uremic syndrome	Pasteurization Strict milking hygiene Farm environmental management Monitoring and diagnosing mastitis. Vaccination, Genetic selection Proper animal nutrition Probiotics *Bacillus subtilis* C-3102; *Saccharomyces cerevisiae*; *L. lactis*; *L. lactis* DPC 3147; *E. mundtii* H81	[85,86,87,92,93,94,95]
MAP Johne’s disease	↑ *Actinobacteria*, ↑ *Proteobacteria*, ↑ *Arthrobacter*, ↑ *Bacillus* ↑ *Camobacterium* ↑ *Desemzia* ↑ *Trichococcus* ↑ *Enterococcus* ↑ *Planococcaceae* ↓ *Bacteroidetes*, ↓ *Firmicutes*, ↓ *Verrucomicrobia*, ↓ *Akkermansia* ↓ *Paraprevotellaceae* ↓ *Faecalibacterium*	Alter lipid metabolism; Granulomatous inflammation of the intestinal mucosa; Malabsorption; Diarrhoea; Weight loss;	↓ milk production; ↑ SCC MAP detected in milk/colostrum.	MAP is associated with Crohn’s disease; Dysbiosis;	Milk testing for MAP; Manure management to reduce MAP (especially when fertilizing soil); Animal control and prevention programs; Effective pasteurization; Animal Vaccination; Upcoming human vaccination; Milk Pasteurization	[75,79,92,96,97,98,99]
ARGs subclinically diseased	↑ *Oxytrichidae*, ↑ *Azospirillum* sp. CAG_239, ↑ *Pseudocohnilembidae* ↑ *Proteobacteria*	Low-intensity chronic inflammation; unstable fermentation ↓ Ruminal pH disturbed Dysbiosis	Presence of (blaCMY-2) ↓ milk texture ↓ milk flavor; Milk ARGs	AMR transfer of ARGs into the human microbiota; Global threat to the environment and public health;	One Health surveillance Reduction in antibiotic use; Targeted probiotics to mitigate ARGs; Manure treatment for reducing ARGs; Early detection of subclinical diseases; Feeding and microbial management practices;	[100,101,102,103,104,105,106,107,108]
*Listeria* *monocytogenes*	Dysbiosis	↑ IL-1β, IL-6 and TNF-α; Fever; Lethargy; Neurological manifestations of listeriosis; Reproductive disorders; Blood poisoning; Mastitis;	Imbalance in milk protein fractions; ↓ casein, ↑ whey proteins; ↓ lactose; ↓ milk production; ↑ SCC	Listeriosis; Risk for pregnant women and immune-suppressed individuals; Nausea; Diarrhoea; Severe systemic infections;	Hygiene and contamination control measures; Implementing biosecurity practices; Systematic surveillance of animal health;	[109,110,111,112,113,114]
*Escherichia coli* *pathogenic*	Dysbiosis ↑ *Proteobacteria*, ↓ *Firmicutes* ↓ *Bacteroidetes* ↑ *Escherichia* ↑ *Shigella* ↓ *Bacteroides*, ↓ *Lactobacillus* ↓ *Blautia*	Mastitis; Sever diarrhea; ↑ IL-1β, IL-6 and TNF-α; Dysbiosis	↑ SCC; Ionic imbalances; ↓ milk quality	Hemorrhagic colitis; HUS; Urinary tract infection; Gastroenteritis; AMR	One Health surveillance; Pasteurisation; Hygiene and contamination control measures; Animal control and prevention; Addition of Chitosan Microparticles to Feed	[115,116,117,118,119]
*Salmonella enterica*	Small and large intestine, ↓ *Clostridiales*, ↓ *Subdoligranulum*, ↓ *Bacteroides*, ↑ *Prevotella*, ↑ *Faecalibacterium*, ↑ *Akkermansia*, ↑ *Enterobacterales*;	↑ IL-1β, IL-6 and TNF-α; Dysbiosis;	↓ milk quality; ↑ SCC; Affects coagulation and cheese production	Gastrointestinal Salmonellosis; acute Gastroenteritis and bacteremia; AMR;	One Health surveillance; host-adapted probiotics; Fermentable fiber–rich diets enhance beneficial bacteria (*Prevotella*, *Lachnospiraceae*, *Ruminococcus*) and reduce *Salmonella*. Detection of bacteria; Sanitation;	[120,121,122]
*Staphylococcus aureus*	↓ *Prevotella*, ↑ *Proteobacteria*, ↑ *Moraxellaceae* ↑ *Stenotrophomonas*	↑ IL-1β, IL-6 and TNF-α; Dysbiosis; Mastitis; Disturbed fermentation;	↓ milk quality; ↑ SCC; milk microbiota changes significantly; ↓ Firmicutes; ↓ Actinobacteria	From superficial skin lesions to life-threatening systemic diseases; MRSA	One Health surveillance; Biosecurity measures; Reduction in antibiotic use; Regular sanitation and rigorous disinfection procedures; Quarantine and genetic analysis to detect strains with interspecies transmission potential	[123,124,125,126,127]
Rumen Acidosis	↓ *Bacteroidetes* ↓ *Fibrobacter succinogenes* ↓ *Ruminococcus albus* ↓ *Ruminococcus bicirculans* ↓ *Butyrivibrio fibrisolvens* ↑ *Prevotella bryantii* ↑ *Selenomonas ruminantium* ↓ *Streptococcus* ↑ *Lactobacillus* ↑ *Succiniclasticum* ↑ *Clostridium*	↓ Ruminal pH disturbed Dysbiosis; Systemic inflammation;	↓ milk quality; ↓ milk production; ↓ lactose; ↓ protein; ↓ fat; ↓ the taste, consistency and clotting ability of milk; ↑ SCC; favoring contamination of milk with pathogenic bacteria;	↑ pathogens, risk of enterotoxins; Sudden diarrhea, nausea, vomiting, cramps;	Balanced diet with a sufficient proportion of structural fibers to stimulate rumination and salivation; Gradual introduction of concentrates to allow the ruminal microbiota to adapt; One Health surveillance.	[92,128]

OTUs, Operational Taxonomic Unit; SCFA, short-chain fatty acids; UFA, unsaturated fatty acids; CLA, conjugated linoleic acid; SCC, somatic cell count; AMR, antimicrobial resistance; IL-1β, Interleukin-1 beta; IL-6, Interleukin-6; TNF-α, Tumor Necrosis Factor alpha; ROS, reactive oxygen species; MAP, *Mycobacterium avium* subsp. *paratuberculosis;* ARGs, Antibiotic resistance genes; blaCMY-2, AmpC-type β-lactamase; HUS, Hemolytic uremic syndrome; MRSA, methicillin-resistant *S. aureus*; ↑ increase; ↓ decrease.

### 3.2. Poultry

The gastrointestinal tract (GIT) of poultry hosts a diverse microbiota, comprising bacteria, archaea, fungi, and viruses [129], which is essential for nutrient absorption and for protecting against pathogenic microorganisms [130]. The intestinal microbiota of poultry is primarily composed of bacteria belonging to the phyla *Firmicutes* (e.g., *Lactobacillus*, *Clostridium*, *Enterococcus*, *Turicibacter* and *Romboutsia*), *Bacteroidetes* and *Proteobacteria* (e.g., *Escherichia coli* and *Helicobacter*), as well as *Actinobacteria* (e.g., *Bifidobacterium*, *Olsenella* and *Collinsella*). The diversity and dynamics of these microbial communities vary depending on the species and age of the birds, as well as environmental conditions [131].

Poultry are exposed to a variety of stressors, including heat and various diseases, which significantly impact their health. Their well-being increasingly relies on the integrated functioning of the immune system and metabolism. The gut microbiota produces important metabolites, such as SCFAs and vitamins, which play crucial roles in energy metabolism, maintaining intestinal barrier integrity, and modulating immune responses. These functions are vital for the immune competence of poultry, energy metabolism, intestinal protection, and, most importantly, their ability to handle stress and disease. As a result, a balanced gut microbiota enhances vaccination effectiveness, enhances resistance to infections, and improves overall production performance. Within the One Health framework, investigating the poultry gut microbiome is of paramount importance. The microbiota plays a central role in maintaining poultry health by regulating digestion and nutrient absorption, suppressing pathogenic bacteria such as *Salmonella*, *Clostridium perfringens*, and *Campylobacter*, and modulating the immune system to enhance disease resistance [132,133,134].

In poultry, dysbiosis may occur as a result of high-concentrate diets, low dietary fiber intake, stress, antibiotic administration, or infectious challenges [135]. This reduces beneficial bacteria (Lactobacillus, Bifidobacterium) and increases pathogenic bacteria, increasing susceptibility to diseases such as necrotic enteritis and salmonellosis. Dysbiosis is also associated with impaired activation of protective T-cell responses, systemic inflammatory responses that can affect growth and feed conversion efficiency.

Gut dysbiosis in poultry not only affects bird health but also has direct consequences on food safety and quality (Table 2) by influencing the risk of contamination in meat and eggs, thereby affecting the transmission of zoonotic pathogens (*Salmonella*, *Campylobacter*, *Clostridium perfringens*) to humans [136,137]. Additionally, research indicates that the poultry microbiome contributes to the nutritional quality (protein, lipid content, vitamin levels) of these products, which are among the most widely consumed foods worldwide [138,139,140]. Maintaining a balanced microbiota is therefore crucial to ensuring safe, high-quality poultry products for human consumption.

Humans can be exposed to pathogenic bacteria through the consumption of contaminated meat, eggs, or other poultry products. These can cause diarrhoea, vomiting, and abdominal cramps from *Salmonella* and enterotoxigenic *E. coli*. Also, *Staphylococcal* food poisoning is caused by enterotoxins produced by *S. aureus* in contaminated food. Potential exposure to AMR bacteria increases the public health risk. Maintaining gut microbial balance is key to both animal health and food safety.

Among the most recommended preventive measures are the inclusion of dietary fiber, prebiotics, and a well-balanced nutrient ratio. Continuous monitoring of intestinal health, growth performance, and feed conversion efficiency is essential for the early detection of dysbiosis or disease. Supplementation with probiotics such as *Lactobacillus* (*L. acidophilus*, *L. plantarum*, *L. casei*, *L. reuteri*, *L. salivarius*), *Bifidobacterium* (*B. bifidum*, *B. longum*), *Enterococcus* (*E. faecium*, *E. faecalis*), *Bacillus* spp., *Saccharomyces cerevisiae*, and *Aspergillus oryzae* has demonstrated beneficial effects on both growth performance and immune function by restoring microbial balance [141,142]. These microorganisms play a key role in reducing intestinal pH, enhancing digestive efficiency, stimulating immune responses, maintaining gut microbiota balance, improving nutrient absorption, and producing digestive enzymes. When these strategies are combined, they create synergistic effects. Furthermore, reducing stress and using antibiotics carefully are essential for preventing the development of intestinal dysbiosis.

**Table 2:** Impact of Poultry Gut Microbiome Disruption: Causes, Effects on Food Products, and Implications for Human Health.

Cause	Microbiome Effects	Immune/Metabolic Changes	Impact on Animal and Products	Human Health Impact	One Health Prevention/Intervention Strategies	Reference
*Staphylococcus aureus*	Dysbiosis	↑ IL-1β, IL-6 and TNF-α	Colonizes the intestinal tract and eggs	Food poisoning; Diarrhea, nausea, vomiting, abdominal cramps;	One Health surveillance; Rigorous hygiene; Probiotics (*Lactobacillus*, *Bifidobacterium*, *Enterococcus*, *Saccharomyces*)	[142]
*Salmonella pullorum*	↓ *Bacteroides*, ↓ *Desulfovibrio*, ↓ *Megamonas*, ↑ *Escherichia-Shigella*	↓ IFN-γ ↓ IL-2 ↑ IL-4	↓ egg production; ↓ fertility; ↓ hatchability;↑ mortality	Mild enteritis AMR	Biosecurity measure Vaccination; Probiotics (*Lactobacillus paracasei Lactobacillus plantarum*);	[129,143,144,145,146,147]
*Salmonella* *Typhimurium*	Dysbiosis ↑ *Enterobacteriaceae* ↑ *Bacteroides* ↓ *Enterococcus*, ↓ *Lactobacillus*, ↓ *Escherichia*, ↓ *Bacillaceae*	Affects systemic muscle metabolism; alters AMPK activity and insulin/mTOR	↓ weight; ↓ digestion ↓ nutrient absorption ↓ safety for meat and eggs	Fever, diarrhoea, and cramping in the abdomen; Meningitis; Blood stream infections;	One Health surveillance; Biosecurity measures; Prudent use of antibiotics to mitigate AMR; Bioactive feed additives; Vaccination;	[148,149,150,151]
*Salmonella Enteritidis*	↑ *Firmicutes* ↓ *Bacteroidetes*	Changes in energy utilization and fat/glucose metabolism (AMPK and mTOR)	↓ weight; ↓ digestion ↓ nutrient absorption ↓ safety for meat and eggs	Fever, diarrhoea, and cramping in the abdomen, meningitis and blood-stream infections;	Vaccination; One Health surveillance; Bioactive feed additives;	[152,153,154,155]
*Campylobacter* spp.	Dysbiosis ↓ *Lactobacillus* ↓ *Clostridium*	pro-inflammatory immune responses, ↑ IL-6, ↑ IL-17A ↑ IL-17F	↓ weight; ↓ digestion ↓ nutrient absorption ↓ safety for meat and eggs	Campylobacteriosis; Diarrhea, fever, abdominal pain; Neurological complications (Guillain-Barré syndrome)	Hand and utensil hygiene; Avoiding the consumption of raw or undercooked meat	[156,157]
*Toxoplasma gondii*	Dysbiosis	Specific antibodies IgM and IgG; ↑ IIFN-γ ↑ TNF-α ↑ IL-6	Forms cysts in the muscles	Toxoplasmosis is usually asymptomatic; dangerous in pregnancy (miscarriage, malformations)	Avoiding the consumption of raw or undercooked meat; Rigorous hygiene;	[158,159]

IFN-γ, Interferon gamma; IL-2, Interleukină 2; IL-4, Interleukină 4; AMPK, Activated Adenosine Monophosphate Kinase; insulin/mTOR, insulin/mammalian target of rapamycin (mTOR); IL-1β, Interleukin-1 beta; IL-6, Interleukin-6; TNF-α, Tumor Necrosis Factor alpha; IL-17A, Interleukin 17A; IL-17F, Interleukin-17F; IgM, Immunoglobulin M; IgG, Immunoglobulin G; ↑ increase; ↓ decrease.

### 3.3. Pig

The intestinal microbiota of pigs is complex and comparable to that of humans. It is dominated by *Firmicutes* (68.65%) and *Bacteroidetes* (20.94%), alongside smaller proportions of *Proteobacteria*, *Spirochaetes*, *Tenericutes*, *Actinobacteria*, *Verrucomicrobia*, *Cyanobacteria*, and *Planctomycetes* [160]. As in other species and humans, the characteristics of the microbiota vary with factors such as diet, age, and gender.

Pigs are widely used as models of the human gut microbiome due to their physiological and immunological similarities and their omnivorous diet [161]. They can also serve as reservoirs for zoonotic pathogens. Occupational exposure on pig farms has been shown to shape the human gut microbiota, with workers exhibiting higher levels of *Prevotellaceae* and lower levels of *Bacteroidaceae*, resembling the porcine intestinal microbial profile [162]. These microorganisms can be inhaled and ingested through the air, thereby altering the human microbiota.

The causes of dysbiosis in pigs include the use of antibiotics, which promote the growth of resistant or opportunistic bacteria [44], inadequate diets or abrupt dietary changes [163], stress resulting from transport, overcrowding, or poor housing conditions, and infections with pathogens [164].

The gut microbiome can influence weight gain and energy metabolism in animals and humans [165]. A recent study investigated the effects of dietary restriction in pigs and analysed which factors influence metabolism through the gut microbiome. The pigs’ gut microbiomes were then transplanted into gnotobiotic mice, allowing them to be evaluated in a controlled environment. The results showed that the gut microbiome directly affects weight gain and energy metabolism, highlighting its central role in regulating the host’s development and metabolic processes [165]. These studies suggest that managing the gut microbiome could support healthy growth in farm animals, especially when dietary restrictions are in place or when antibiotics are not used to promote growth. Similarly, these mechanisms have implications for humans, suggesting that microbiome optimisation could be beneficial in cases of malnutrition or suboptimal diets.

In pigs, dysbiosis has been associated with variations in meat quality traits, including intramuscular fat content and the physicochemical characteristics of muscle tissue [166].

Another relevant approach to studying the human microbiota is to investigate changes in the gut microbiota of pigs in the context of infectious diseases, analyzing the impact on intestinal barrier integrity and local immune responses in the presence of pathogens [167]. In pigs infected with pathogens such as *Mycoplasma hyorhinis*, dysbiosis of the gut microbiota is associated with reduced expression of tight junction proteins, increased markers of barrier dysfunction and systemic inflammation, indicating that infection not only alters microbial communities but also compromises intestinal integrity and immune homeostasis [164]. Dysbiosis in pigs can significantly affect both animal production and the quality of their products (Table 3). From a zoonotic and public health standpoint, dysbiosis in pigs can enhance susceptibility to pathogens with recognized zoonotic potential, such as Salmonella and *Brachyspira pilosicoli*. Imbalances in microbial communities may lead to the overgrowth and shedding of these bacteria, increasing the risk of contaminating meat products, farm environments, and water sources, thereby increasing the risk of human exposure [168]. Measures for the prevention and mitigation of dysbiosis in pigs include a balanced diet and nutritional strategies, supplementation with prebiotics and probiotics (Lactobacillus, Bifidobacterium) to support epithelial health, the competitive exclusion of pathogens, and good management practices aimed at improving hygiene and reducing stressors [169].

**Table 3:** Impact of Pig Gut Microbiome Disruption: Causes, Effects on Food Products, and Implications for Human Health.

Cause	Microbiome Effects	Immune/Metabolic Changes	Impact on Animal and Products	Human Health Impact	One Health Prevention/Intervention Strategies	Reference
*Trichinella spiralis*	Dysbiosis	Immune responses The infection alters the Th2 response and the activity of LP Treg cells. Production of specific antibodies IgA and IgG1;	Intestinal inflammation; Diarrhea; ↓ Growth performance ↓ animal welfare	Trichinosis, fever, muscle aches, facial swelling, abdominal pain, diarrhea	One Health surveillance; Pig nutrition control; Regular deworming of pigs; Meat test for *trichinella*; Freezing meat −15 °C for 20 days; Cooking meat at least 71 °C; Rigorous hygieneș;	[170,171,172]
*Taenia solium*	Dysbiosis	Axonal and neuronal damage; Alteration of the permeability of the blood–brain barrier, which allows the influx of peripheral immune cells (eosinophils, macrophages, B and T lymphocytes, plasma cells)	Dullness, sluggishness, somnolence, apathy; Local inflammation in muscle tissue;	Taeniasis, Human cysticercosis, Neurocysticercosis, Altered appetite, abdominal pain, nausea, diarrhea or constipation, weight loss	One Health surveillance; Rigorous hygiene; Rigorous inspection of the carcass Vaccination	[173,174,175,176,177]
*Streptococcus suis*	Dysbiosis	↑ TNF-α ↑ IL-6 ↑ IL-1β B lymphocyte activation;	Meningitis, Septicemia, Pneumonia, Polyarthritis, Endocarditis ↓ appetite; Abnormal postmortem pH; ↓ carcasses, ↓ yield, ↓ processing chain.	Fatal infections, including septicemia and meningitis	One Health surveillance; Diagnostic marker genes	[178,179,180]
HEV	Dysbiosis	↑ T lymphocytes (Treg) ↓ CD4^+^CD8^+^ T lymphocytes ↑ ALT, ↑AST	Liver inflammation; Activation of humoral immunity with the production of neutralizing anti-HEV antibodies	Hepatitis E	One Health surveillance; Zoonotic control (porcine HEV) Vaccination	[181,182]

HEV, Hepatitis E virus; IgA, Immunoglobulin A; IL-1β, Interleukin-1 beta; IL-6, Interleukin-6, TNF-α, Tumor Necrosis Factor alpha; ↑ increase; ↓ decrease.

### 3.4. Fish

The GIT microbiome of fish differs from that of other vertebrates, being dominated by *Proteobacteria* (≈51.7%) and *Firmicutes* (≈13.5%) [183]. The gut microbiota is influenced more strongly by the host’s habitat (freshwater vs. marine) than by phylogenetic affiliation or diet [183]. Thus, freshwater fish are characterized by a predominance of the phyla *Firmicutes* and *Fusobacteria*, particularly the genus *Cetobacterium*, whereas marine fish are dominated by the phylum *Proteobacteria*. Within the class *Gammaproteobacteria*, marine species are mainly associated with the families *Moraxellaceae*, *Vibrionaceae*, and *Enterobacteriaceae*, along with *Alcaligenaceae* (*Betaproteobacteria*), while freshwater fish are enriched in *Aeromonadaceae* (*Gammaproteobacteria*). Moreover, *Clostridiaceae* (*Clostridia*) exhibited higher abundance in freshwater fish compared to marine counterparts, whereas *Leuconostocaceae* (*Bacilli*) is more abundant in marine fish [183]. The dynamic microbiome enables fish to adapt to their specific environment by modulating physiological and metabolic functions [184].

However, exposure to various stressors such as antibiotic treatments, extreme temperature fluctuations, bacterial infections, parasitic infestations, and aquatic environmental pollutants can disrupt the intestinal microbiota, leading to dysbiosis. Microbiome imbalance impairs the production of beneficial metabolites (SCFA), compromising intestinal barrier integrity and growth performance in fish, particularly in intensive aquaculture systems.

Fish and fishery products, as well as aquatic organisms, are considered healthy due to their high biological value [184]. However, caution is required, as they also represent one of the food groups with the highest risks if they are not fresh, properly processed, or if they may contain toxins [185].

A recent study shows that the intestinal microbiome of marine fish harbors a diversity of bacteria capable of producing bacteriocins, which may be used in the development of new antibiotics to combat drug-resistant bacterial infections [186]. Another important aspect is that fish consumption, particularly of cod or salmon, can modulate the human gut microbiome, thereby contributing to intestinal health [187].

The consumption of fish, particularly fatty species, provides a beneficial source of Eicosapentaenoic Acid (EPA) and Docosahexaenoic Acid (DHA), which can promote the growth of beneficial bacteria (e.g., *Bifidobacterium*, *Lactobacillus*) as well as short-chain fatty acid (SCFA) producing bacteria, contributing to the maintenance of intestinal barrier integrity, regulation of inflammation, and modulation of immune responses [188].

Details on the impact of the fish microbiome and zoonoses on human health through food products are presented in Table 4.

## 4. Linking Animal-Derived Foods to Gut Microbiome Dynamics and Human Health Implications

Recent technological advances in the field of ‘omics’ have profoundly transformed the study of microbiota. The use of 16S and 18S rRNA gene sequencing has enabled microbes to be classified with greater accuracy, from the phylum level to the species level [203], whereas traditional culture methods only allowed for the classification of less than 1% of human microorganisms [203,204]. Recently, on these tools, a global mapping of the human gut microbiome was achieved by analyzing 32,152 metagenomes from 94 microbiome studies, revealing the extensive genetic diversity and complexity of the microbial communities inhabiting the human gastrointestinal tract [205].

These insights provide a foundation for the integration of microbiome science into strategies for the prevention of zoonotic diseases [30,206]. Microorganisms present in humans, animals, plants, and the environment are critical for maintaining ecosystem health through co-evolution and co-regulation [29], for disease control via pathogen suppression [207,208], for influencing antimicrobial resistance (AMR) [209], and for supporting environmental resilience and nutrient cycling [210].

Within this framework, studies increasingly highlight the complex relationship between the consumption of animal-derived foods, gut microbiome dynamics, and human health. Animal proteins (meat, dairy, eggs) exert distinct influences on the microbiome, and differences among types of meat and dairy products can modulate gut health in diverse ways. For example, processed red meat may reduce beneficial short-chain fatty acid (SCFA) producing bacteria, whereas fermented dairy products support microbial diversity [45]. Another important aspect is that animal proteins can stimulate the production of trimethylamine (TMA) in the microbiome, which is subsequently metabolized into trimethylamine N-oxide (TMAO), a compound associated with cardiovascular risks [211].

Fermented dairy products (yogurt, cheese, kefir, etc.) can positively influence the gut microbiome through their probiotic cultures, which can support microbial balance and reduce the risk of IBD [45]. Table 5 presents a detailed overview of the impact of dietary factors on the human microbiome.

The human microbiome represents a highly complex community of microorganisms inhabiting both internal and external surfaces of the human body, exerting critical influences on health and disease [219]. Within the gut, *Firmicutes* and *Bacteroidetes* dominate, together accounting for nearly 90% of the microbial population, while *Actinobacteria*, *Fusobacteria*, *Proteobacteria*, and *Verrucomicrobia* are present at lower abundances [220]. These microbial groups play essential roles in nutrient digestion, the synthesis of vitamins (B and K) [221] and the regulation of immune responses [222]. Beyond bacteria, the human microbiome encompasses a broader diversity of organisms, including Archaea [223], fungi, viruses, protozoa, and parasites [224].

Numerous studies have identified specific microbial taxa that are consistently altered in the gut microbiota of patients with IBD and celiac disease, suggesting potential roles in disease pathogenesis and immune modulation. In IBD, dysbiosis is characterized by a reduced abundance of beneficial SCFA producers such as *Faecalibacterium prausnitzii*, Roseburia spp., members of *Lachnospiraceae*, and *Ruminococcaceae*, alongside an increased prevalence of pro-inflammatory *Proteobacteria*, including adherent invasive *Escherichia coli* and members of the *Enterobacteriaceae* family, which have been linked with disease activity and mucosal immune responses [225]. Similarly, in celiac disease, patients exhibit gut microbiota alterations with increased representation of *Bacteroides* and *Escherichia coli*, and decreased levels of commensal genera such as *Lactobacillus* and *Bifidobacterium*, which may contribute to barrier dysfunction and immune activation in genetically susceptible hosts [226]. These microbial alterations, especially the reduction in anti-inflammatory taxa and enrichment of pathobionts, underscore the immunomodulatory potential of the microbiome in chronic intestinal inflammation and highlight the need for further mechanistic studies to establish direct cause–and–effect relationships.

Microbial transfer between humans and animals can occur either directly or indirectly. Direct transfer occurs through close contact with animals, including handling, caregiving, or consumption of animal-derived products (meat, milk, eggs), facilitating the transfer of microorganisms between species [227]. These interactions can influence the diversity and composition of the human microbiome, with potential health implications. Indirect transfer occurs via contamination of soil with uncomposted or insufficiently treated manure, which may contain *Escherichia coli* and *Salmonella* [228]; through contamination of irrigation water [229]; and via farm bioaerosols and dust, which can be transported over long distances, representing a significant route for zoonotic disease transmission [230].

Although there is a substantial body of literature on dysbiosis in animals and humans, very few studies investigate direct cause–effect relationships within the One Health framework, tracing pathways from environmental sources (soil, water, air) to animal-derived products and ultimately to human health outcomes. The available information is extensive but fragmented, which limits the ability to draw comprehensive conclusions. Additional research is needed to cover a wider range of pathologies, species, and environmental contexts to develop an integrated understanding of microbiome-related health impacts across the One Health continuum.

## 5. Conclusions

The gut microbiome of farm animals and aquatic species has a major impact on animal health, productivity, food safety, and immune and metabolic processes, with direct consequences for human health. Across a wide range of animals, from ruminants to monogastrics and fish, dysbiosis commonly arises in response to nutritional imbalances, environmental stressors, antimicrobial exposure, and infections. This can influence pathogen shedding, the emergence of antimicrobial resistance, and the quality of animal-derived food products.

Despite the large volume of literature on dysbiosis in animals and humans, important research gaps remain. Most studies are cross-sectional, species-specific, and focused on isolated components of the One Health continuum, which limits the ability to establish direct cause–and–effect relationships. Few studies trace microbiome changes from environmental reservoirs (soil, water, air) through food-producing animals and aquaculture systems to animal-derived products, and then to effects in the human gut microbiome, immune function, and disease risk. Moreover, variation in study methodologies, host species, diets, and environments makes it difficult to compare results and understand underlying mechanisms.

This review is unique in introducing a framework that highlights connections among microbes across species, diets, and the immune system. By integrating data from terrestrial livestock, ruminants, poultry, pigs, fish, and humans, it illustrates how microbial, immunological, and metabolic pathways collectively influence zoonotic risks, food safety, and susceptibility to chronic disease. Rather than treating animals’ and human microbiomes as separate, this synthesis emphasizes their biological continuity across environmental, nutritional, and production systems. It provides a conceptual foundation for future longitudinal, multi-omics, and interdisciplinary research aimed at microbiome-informed disease prevention and sustainable health management.

## Figures and Tables

**Table 4:** Impact of Fish Gut Microbiome Disruption: Causes, Effects on Food Products, and Implications for Human Health.

Cause	Microbiome Effects	Immune/Metabolic Changes	Impact On Animal And Products	Human Health Impact	One Health Prevention/Intervention Strategies	Reference
*Clostridium botulinum*	Dysbiosis	↑ TNF-α) ↑ IL-1β IgM antibodies	Loss of equilibrium and motion, abducted opercula, open mouths, dark pigmentation, and head up/tail	Muscle weakness, blurred vision, difficulty swallowing, flaccid paralysis, respiratory failure	One Health surveillance; Appropriate thermal processing, Specific amplification product PCR with the most probable number (MPN) method, Aquatic environment control, Strict refrigeration, Hygienic handling, Regular monitoring;	[189,190]
*Anisakis* spp.	Dysbiosis	Activation of phagocytes, macrophages, and neutrophils; ↑ TNF-α ↑ IL-1β Leukocyte infiltration;	Presence of Anisakis larvae in the fish muscle, often without obvious clinical signs;	Anisakiasis, Acute abdominal pain, nausea, vomiting, and, in more severe cases, intestinal perforation;	Freezing the fish at temperatures of −20 °C for at least 24 h, Visual testing or inspection, Quick cleaning and evisceration, and HACCP procedures.	[191,192]
*Mycobacterium* *marinum*	Dysbiosis	Activation of phagocytes, macrophages, and neutrophils; ↑ TNF-α ↑ IL-1β Granuloma formation; Chronic inflammation;	Skin lesions, ulcers, and inflammation of the skin	Cutaneous mycobacteriosis Granulomatous skin lesions, subcutaneous nodules, arthritis, osteomyelitis	One Health surveillance Quarantine and testing of the new batch, Disinfection, and water quality control	[191,193]
*Aeromonas hydrophila*	Dysbiosis	↑ TNF-α ↑ IL-1β IgM antibodies	Skin lesions, ulcers, and inflammation of the skin; Affects tissues and blood vessels	Aeromoniasis Gastroenteritis, skin infections, septicemia	One Health surveillance; Maintaining optimal water conditions; Administration of bacteriophages; PCR test; Monitoring and control of antibiotic use;	[194,195]
*Edwardsiella tarda*	Dysbiosis	↑ TNF-α ↑ IL-1β	Skin lesions, ulcers, and inflammation of the skin	Edwardsielloza Gastroenteritis Arthritis Septicemia Meningitis	One Health surveillance; Upcoming vaccination; Avoiding the consumption of raw or undercooked fish	[196,197,198,199]
*Vibrio* spp.	Dysbiosis	↑ TNF-α ↑ IL-1β Local and systemic inflammation IgM production	Skin lesions, ulcers, and inflammation of the skin	Vibriosis, Diarrhoea, abdominal cramps, fever, chills, sepsis, and skin lesions;	One Health surveillance, Avoiding the consumption of raw or under-cooked fish, Vaccination	[200,201,202]

TNF-α, Tumor Necrosis Factor alpha; IL-1β, Interleukin-1 beta; IgM-Immunoglobulin M; HACCP, Hazard Analysis and Critical Control Points; ↑ increase; ↓ decrease.

**Table 5:** The impact of diet on the human microbiome.

References	N	Diet/Nutrition	Age	Gender	Microbiome Composition/Associated Bacteria
[212]	40	Meat	18–30	male	Pork consumption: ↑ *Firmicutes*, ↓ *Bacteroidetes*, Genus: ↑ *Bacteroides*, Chicken consumption: ↓ *Firmicutes*, ↑ *Bacteroidetes*, Genus: ↑ *Prevotella*, ↑ *Dialister*, ↑ *Faecalibacterium*, ↑ *Olsenella*
[213]	59	Fried meat	18–35	-	↓ *Lachnospiraceae*, ↓ *Flavonifractor*, ↑ *Dialister*
	58	Control grup			↑ *Dorea*, ↑ *Veillonella*
[214]	19.817	Omnivores	-	male and female	↑ *Ruminococcus torques*, ↑ *Bilophila wadsworthia*, ↑ *Alistipes putredinis*
	656	Vegans	-		↑ *Lachnospiraceae*, ↑ *Butyricicoccus* sp., ↑ *Roseburia hominis*
	1.088	Vegetarians	-		↑ *Streptococcus thermophilus*, ↑ *Lactobacillus rhamnosus*, ↑ *L. delbrueckii*, ↑ *L. paracasei*, ↑ *L. lactis*, ↑ *L. acidophilus*
[215]	10	Control grup	25–63	male and female	↓ *Eggerthella*, ↓ *Ruminiclostridium* 9, ↓ *Sellimonas*, ↓ *Akkermansia*
	14	Yogurt			↑ *Akkermansia*, ↑ *Ruminococcaceae* UCG-002, ↑ *Alistipes*, ↑ *Erysipelatoclostridium*, ↑ *Sellimonas*, ↑ *Barnesiella*, ↑ *Eggerthella*, ↑ *Flavonifractor*, ↑ *Oscillibacter*, UBA1819, ↑ *Ruminiclostridium 9* ↓ *Megasphaera*
	9	Yogurt + Hot Spring			↓ *Lachnoclostridium*, ↓ *Holdemania*
[216]	13	kefir	18–30	-	↑ *Bifidobacterium breve*, ↑ *Pararoseburia lenta*, *↑ Akkermansia massiliensis*, ↑ *Lactococcus lactis*, ↑ *Weissella koreensis*, ↑ *Ruthenibacterium lactatiformans*, ↑ *Leuconostoc mesenteroides*, ↓ *Streptococcus thermophilus*, ↓ *Streptococcus gallolyticus*, ↓ *Anaerobacterium chartisolvens*, ↓ *Weissella confusa*, ↓ *Anaerotignum faecicola*, ↓ *Massiliomicrobiota timonensis*
	9	milk			↑ *Waltera intestinalis*, ↑ *Enterococcus faecium*, ↑ *Ruthenibacterium lactatiformans*, ↑ *Faecalibacillus faecis*, ↑ *Anaerobacterium chartisolvens*, ↑ *Alistipes finegoldii*, ↑ *Massiliomicrobiota timonensis*, ↑ *Leuconostoc mesenteroides*, ↓ *Sutterella wadsworthensis*, ↓ *Streptococcus thermophilus*
	6	yogurt			↑ *Streptococcus thermophiles*, ↓ *Pediococcus inopinatus*
[217]	30.154	White meat	50–65	male and female	↑ *Streptococcus australis*, ↑ *Veillonella rogosae*, ↑ *Roseburia intestinalis*, ↑ *Ruminococcus* sp., ↑ *Phascolarctobacterium faecium*, ↑ *Eubacteriales* sp., ↓ *Bifidobacterium longum* subsp. *longum*, ↓ *Bifidobacterium adolescentis*, ↓ *Lachnospiraceae*, ↓ *Sutterella wadsworthensis*, ↓ *Oscillospiraceae*.
		Processed red meat			↑ *Latilactobacillus sakei* subsp. *Sakei*, ↑ *Ruminococcus torques Blautia obeum*, ↑ *Dorea formicigenerans*, ↑ *Veillonella rogosae*, ↑ *Eubacteriales* sp., ↑ *Eggerthellaceae* sp., ↓ *Clostridium* sp., ↓ *Oscillospiraceae*, ↓ *Rothia mucilaginosa*, ↓ *Lachnospiraceae*.
		Unprocessed red meat			↑ *Amedibacillus dolichus*, ↑ *Coprococcus comes*, ↑ *Latilactobacillus sakei* subsp. *sakei*, ↑ *Eubacteriales* sp., ↓ *Streptococcus mutans*, ↓ *Lachnospiraceae*, ↓ *Bifidobacterium*
[218]	16	lean red meat	20	-	↑ *Bacillota*, ↑ *Actinomycetota*, ↓ *Bacteroidota*, ↓ *Chloroflexota*, ↓ *Pseudomonadota*, ↓ *Synergistota*
		lean white meat			↑ *Bacillota*, ↓ *Actinomycetota*, ↓ *Bacteroidota*, ↓ *Chloroflexota*, ↓ *Pseudomonadota*, ↓ *Synergistota*

N, Number of participants; ↑ increase; ↓ decrease.

## Data Availability

No new data were created or analyzed in this study. Data sharing is not applicable to this article.

## Abbreviations

The following abbreviations are used in this manuscript:

HDACs	Histone deacetylases
IBD	Inflammatory bowel disease
Tregs	Regulatory T cells
Th17	T helper 17 cells
VFAs	Volatile fatty acids
GIT	Gastrointestinal tract
CO2	Carbon dioxide
ARGs	Antibiotic resistance genes
AMR	Antimicrobial resistance
MAP	Mycobacterium avium subsp. paratuberculosis
MRSA	Methicillin-resistant S. aureus
Th2	T helper 2
LP	Lamina propria
ALT	Alanine Aminotransferase
AST	Aspartate Aminotransferase
EPA	Eicosapentaenoic Acid
DHA	Docosahexaenoic Acid
SCFA	Short-chain fatty acids
PCR	Polymerase Chain Reaction,
MPN	Most probable number
TMA	Trimethylamine
TMAO	Trimethylamine N-oxide
OTUs	Operational Taxonomic Unit
UFA	Unsaturated fatty acids
CLA	Conjugated linoleic acid
SCC	Somatic cell count
IL-1β	Interleukin-1 beta
IL-6	Interleukin-6
TNF-α	Tumour Necrosis Factor alpha
ROS	Reactive oxygen species
TBARS	Thiobarbituric Acid Reactive Substances
Th1	T helper 1 cells
blaCMY-2	AmpC-type β-lactamase
HUS	Hemolytic uremic syndrome
IFN-γ	Interferon gamma
IL-2	Interleukină 2
IL-4	Interleukină 4
AMPK	Activated Adenosine Monophosphate Kinase
insulin/mTOR	Insulin/mammalian target of rapamycin (mTOR)
IL-17A	Interleukin 17A
IL-17F	Interleukin-17F
IgM	Immunoglobulin M
IgG	Immunoglobulin G
HEV	Hepatitis E virus
IgA	Immunoglobulin A
IgG1	Imunoglobulina G1
HACCP	Hazard Analysis and Critical Control Points
